# The screening tool of behavioral self-regulation “Head-Toes-Knees-Shoulders” task: a preliminary adaptation to Greek older adults

**DOI:** 10.3389/fpsyg.2026.1891008

**Published:** 2026-08-05

**Authors:** Eleni Rachanioti, Dimitra Magkavila, Olga Fragkomichelaki, Magda Dinou, Effie Katsadima, Theodora Foti, Anastasia Tzalla, Anna Dougali, Christina Bourmpou, Maria Staikopoulou, Aphrodite Papantoniou, Fotini Varlami, Irene Dinaki, Maria Tsiountala, Konstantinos Staikopoulos, Tryfon Mavropalias, Efthymia Efthymiou, Demetra Tomprou, Maria Georgiadi, Dimitra Katsarou, Georgios Kougioumtzis, Ioanna Giannoula Katsouri, Harilaos Zaragas, Despina Moraitou, Maria Sofologi, Georgia Papantoniou

**Affiliations:** 1 Laboratory of Psychology, Department of Early Childhood Education, School of Education, University of Ioannina, Ioannina, Greece; 2 Department of Education, School of Education and Social Sciences, Frederick University, Nicosia, Cyprus; 3 Faculty of Arts and Sciences, Division of Social Science, Department of Psychology, Yale University, New Haven, CT, United States; 4 Department of Computer, Informatics and Telecommunications Engineering, International Hellenic University, Serres, Greece; 5 Department of Primary Education, School of Education, University of Western Macedonia, Florina, Greece; 6 College of Interdisciplinary Studies, Zayed University, Dubai, United Arab Emirates; 7 Department of Psychology, University of Crete, Rethymno, Greece; 8 Department of Preschool Education Sciences and Educational Design, University of the Aegean, Rhodes, Greece; 9 Department of Turkish Studies, National and Kapodistrian University, Athens, Greece; 10 Department of Occupational Therapy, University of West Attica, Athens, Greece; 11 Pedagogy and Teaching Methodology Laboratory, Department of Early Childhood Education, School of Education, University of Ioannina, Ioannina, Greece; 12 Laboratory of Psychology, Section of Experimental and Cognitive Psychology, School of Psychology, Aristotle University of Thessaloniki, Thessaloniki, Greece; 13 Laboratory of Neurodegenerative Diseases, Center for Interdisciplinary Research and Innovation (CIRI—AUTH) Balkan Center, Aristotle University of Thessaloniki, Thessaloniki, Greece; 14 Department of Education, Philips University, Nicosia, Cyprus

**Keywords:** affect, behavioral self-regulation, executive functions, general intelligence, older adults, psychometric properties

## Abstract

The aim of the present study was to examine the psychometric properties of the Head-Toes-Knees-Shoulders (HTKS) behavioral self-regulation (executive function) measure in a sample of Greek older population. Specifically, 88 cognitively healthy Greek older adults over the age of 65 participated in the study. The Greek version of HTKS was administered along with the Greek versions of the Mini-Mental State Examination (MMSE), the Raven's Educational CPM, the Number Series (NS) test, the Positive and Negative Affect Scale (PANAS), the Barratt Impulsiveness Scale (BIS-11) and the Impulsive Behavior Scale (UPPS-P). Exploratory factor analysis supported the unidimensional structure of the HTKS. The internal consistency reliability was evaluated with Cronbach's alpha coefficient and found to be acceptable (α = 0.79). Furthermore, significant positive correlations of the HTKS with measures of cognitive ability (CPM, NS, and MMSE) supported its convergent validity. No significant bivariate correlations of the HTKS were found with older adults' emotional state or impulsivity. The path analysis indicated that nonverbal cognitive ability (CPM) and general cognitive ability (NS) positively influenced the performance on HTKS. In turn, behavioral self-regulation (HTKS) was found to influence positively both positive and negative affect as measured by the PANAS. Additionally, nonverbal cognitive ability (CPM) was found to negatively predict negative affect.

## 1 Introduction

Self-regulation can be broadly defined as a dynamic process whereby individuals establish a desired goal and engage in purposeful actions toward its attainment, while concurrently monitoring their progress (Inzlicht et al., 2021). This process entails the capacity to observe, reflect upon, and adjust one's cognitions, emotions, and behaviors in response to contextual demands (McClelland et al., 2018). Furthermore, it involves the ability to initiate and inhibit actions, as well as to regulate the intensity, frequency, and persistence of both verbal and physical behaviors in a manner that is contextually appropriate and consistent with socially acceptable norms (Bodrova and Leong, 2006). Higher levels of self-regulation have been consistently associated with a wide range of positive life outcomes, including enhanced school readiness, improved academic achievement, the adoption of health-promoting behaviors, and better physical and mental health (Pandey et al., 2018). In contrast, deficits in self-regulatory capacity have been linked to a variety of detrimental outcomes, such as engagement in health-risk behaviors, the development of psychological disorders, substance dependence, criminal involvement, and increased likelihood of unemployment (Bogg and Roberts, 2004).

There are two major competing theories concerning the structure of self-regulation. According to the first theory, self-regulation is considered a general capacity without clear differentiation into distinct components such as emotional and behavioral self-regulation (Berkman et al., 2012; Muraven and Baumeister, 2000). In contrast, the second and more prominent theory —adopted in the present work— conceptualizes self-regulation as a multidimensional construct comprising emotional, cognitive, and behavioral components (Cicchetti and Tucker, 1994; McClelland et al., 2010).

Behavioral self-regulation is conceptualized as the deployment of executive functions within the context of an individual's everyday behavioral actions (McClelland and Cameron, 2011; Ponitz et al., 2009). Executive functions are commonly divided into two primary categories: cold executive functions and hot executive functions. This classification reflects the extent to which cognitive processes are influenced by emotional or motivational factors (Nemeth and Chustz, 2020). The cognitive mechanisms underpinning behavioral self-regulation —commonly referred to as cold executive functions— include cognitive flexibility, working memory, and inhibitory control. Specifically, these functions encompass the capacity to shift perspectives and adapt flexibly to changing task demands (cognitive flexibility), to retain and manipulate relevant information (working memory), and to suppress automatic or impulsive responses in favor of goal-directed behavior, particularly in problem-solving contexts (inhibitory control) (Barkley, 2012). Hot executive functions are engaged in situations involving emotional and motivational stimuli, facilitating behaviors that depend on emotional awareness, emotional regulation, empathy, and broader social cognitive processes (Nemeth and Chustz, 2020). Although hot and cold executive functions are theoretically distinguishable, they operate in a highly interdependent manner. Cold executive functions, including working memory and cognitive flexibility, contribute to processes such as emotion recognition and social inference, which are central to hot executive functioning. In turn, impairments in cold executive functions may compromise goal-directed behavior, increasing susceptibility to impulsive actions influenced by emotionally driven (hot) processes. Together, both domains of executive functioning are essential for adaptive functioning in everyday life, particularly in relation to social interaction and emotional regulation (Yeung et al., 2024).

Historically, research on behavioral self-regulation has predominantly focused on children and adolescents, primarily due to its robust predictive validity for a range of developmental outcomes, including academic achievement, social functioning, and mental health (McClelland et al., 2007). In the context of older adulthood—typically defined as the stage beginning at the age of 65—behavioral self-regulation is closely associated with the strategic processes involved in goal setting, goal pursuit, emotional regulation, and the management of impulsive behavior, all of which critically influence the individual's motivational dynamics (Idowu et al., 2024). Declines in self-regulatory capacity are commonly linked to reductions in frontal lobe integrity, declines in executive functioning as a function of age (Bouazzaoui et al., 2014; Idowu and Szameitat, 2023; Jurado and Rosselli, 2007; McCalister and Schmitter-Edgecombe, 2013) and neuropathological cognitive impairment, such as dementia (Cadar, 2018; Mortamais et al., 2017; Zelazo et al., 2004).

Cognition in adulthood is multidimensional, meaning it includes different abilities that change in different ways over time. While some cognitive abilities decline with age, others remain stable or even improve (Gavelin et al., 2021). Baltes (2003) distinguished between two major components of cognition: (a) cognitive mechanics and (b) cognitive pragmatics. Cognitive mechanics refer to the biological “hardware” of the mind. These include basic processes such as speed and accuracy of sensory input, attention, memory (especially visual and motor), discrimination, comparison, and categorization. Because these abilities are strongly influenced by biology, heredity, and overall health, they tend to decline with age. Research suggests that this decline may begin as early as midlife (Salthouse, 2009). Cognitive pragmatics represent the culturally shaped “software” of the mind. These include skills such as reading and writing, language comprehension, educational attainment, professional expertise, and life knowledge that supports coping and self-understanding. Since these abilities are strongly influenced by cultural experience and learning, they can continue to develop and even improve into older adulthood —at least until very advanced age. This distinction closely parallels the difference between fluid intelligence (similar to cognitive mechanics) and crystallized intelligence (similar to cognitive pragmatics) (Santrock, 2024). The decline of cognitive mechanics in late adulthood includes slower processing speed (Salthouse, 2019), reduced working memory capacity (Kronovsek et al., 2021) and a diminished ability to suppress irrelevant information (inhibition) (Davison et al., 2022).

Individual differences in processing speed are closely linked to physical aging (Liu-Ambrose et al., 2021) and it has been identified as a key indicator of daily functional competence in older adulthood (Roye et al., 2022). Additionally, faster processing speed has been associated with greater life satisfaction among adults ages 40–85 (Siedlecki et al., 2020). Changes in attention are also a significant component of cognitive aging (Chen et al., 2021). Studies indicate that older adults have more difficulty than younger adults in filtering out distracting information, and this difficulty increases as tasks require greater attentional effort (Ziegler et al., 2018). Regarding working memory, research indicates that decline begins in the mid-30s and becomes more pronounced after age 66, affecting both verbal and visuospatial components (D'Antuono et al., 2022). These age-related changes are commonly attributed to reduced inhibitory control and greater distractibility in older adults (Davison et al., 2022).

Additionally, executive function skills are increasingly recognized as contributing not only to cognitive performance but also to health, emotion regulation, coping with life challenges, motivation, and social functioning (Ong et al., 2022). Positive affect refers to an individual's subjective emotional state and the degree to which they experience pleasurable engagement with their surroundings. Individuals with high positive affect typically feel enthusiastic, energetic, and alert, whereas those with low positive affect are more likely to experience sadness, low energy, and reduced engagement. In contrast, negative affect reflects the extent to which individuals experience distress and unpleasant emotions, including guilt, fear, and nervousness (Watson et al., 1988). Research indicates that stronger executive functioning in older adults enhances their perceived control over obstacles that hinder goal attainment, which is associated with greater life satisfaction and positive affect (Toh et al., 2020; Wyman et al., 2022; Yagi et al., 2020). However, this evidence is not uniform. Although aging is associated with declines in inhibitory control, working memory and cognitive flexibility, on the other hand emotional experience and regulation tend to follow a more complex trajectory and are not reducible to cognitive performance (Isaacowitz, 2022). Berk et al. (2017) examined whether positive affect (PA) measured by PANAS was associated with changes in cognitive function over 12 years in an adult population sample. They found no significant longitudinal association between PA scores and memory, executive functioning, or information processing speed at the 6- and 12-year follow-up. A cross-lagged study also showed that executive functions predicted eudaimonic well-being in older adults over 9 years, but not in middle-aged adults, suggesting an age-limited effect rather than a general pattern (Lau and Yang, 2025). Another study reported that inhibition was associated with only one well-being facet —personal growth— whereas working memory and flexibility did not predict that outcome, indicating a domain-specific rather than broad executive-function effect (Fusi et al., 2022). In addition, some older adults with early cognitive impairment still reported positive affect, purpose, and life satisfaction despite experiencing higher negative affect, suggesting that poorer cognition does not necessarily eliminate positive emotional well-being (Zhang et al., 2024). Additionally, self-control efforts may temporarily raise negative affect. Wenzel et al. (2024) found that resisting a pleasurable desire (an inhibitory self-control act) led to significantly increased subsequent negative affect. Older adults often face age-related health challenges that necessitate adjustments to daily routines and lifestyle quality. These physical limitations are frequently accompanied by psychological difficulties, including feelings of guilt or regret regarding diminished capabilities or uncontrollable life circumstances. Such experiences can contribute to a more negative appraisal of one's overall life narrative. In this regard, behavioral self-regulation functions as a key psychological resource, enabling individuals to navigate these challenges and continue striving toward meaningful personal goals (Wrosch et al., 2006). This process typically involves the intentional allocation of cognitive and emotional resources, as well as the active pursuit of informational and emotional support, with the aim of sustaining motivation and adaptive functioning. Empirical studies have consistently underscored the importance of self-regulation in late adulthood, particularly in relation to coping mechanisms, behavioral interventions, and the adoption of health-promoting behaviors that support the maintenance or enhancement of physical and mental well-being (McClelland et al., 2018).

## 2 The present study

The Head-Toes-Knees-Shoulders (HTKS) and the HTKS-Revised are measures of behavioral self-regulation for children ages 4–8 (McClelland et al., 2007, 2014; Gonzales et al., 2021), capturing three main aspects of EF (cognitive flexibility, working memory, and inhibitory control) in a game-like activity. The recently revised version of the task (the HTKS-R) was developed to improve the variability of the scores and reduce floor effects (Gonzales et al., 2021; McClelland et al., 2021).

Developed by McClelland et al. (2007), the HTKS requires children to perform the opposite of the examiner's verbal command —for instance, touching their toes when instructed to touch their head. The task is structured in three parts of increasing complexity, each imposing greater cognitive demands. These demands involve the inhibition of automatic motor responses (Nigg, 2000), the ability to shift attention between rule sets (Morra et al., 2018), and the maintenance of task-relevant information in working memory (Garon et al., 2008). Unlike more traditional assessments of executive function, the HTKS is engaging, brief (approximately 5–7 min), and requires no specialized materials, making it particularly well-suited for use in field settings and large-scale studies (McClelland et al., 2007, 2014; Ponitz et al., 2008, 2009).

Previous research using the HTKS has been limited to children between the ages of 4 and 6 (e.g., McClelland et al., 2007; Ponitz et al., 2009). In a longitudinal study, McClelland et al. (2014) examined the psychometric properties of the HTKS and found that, across diverse groups of young children, the measure showed strong construct validity in relation to attention, inhibitory control, and working memory, as well as high internal consistency reliability. A number of studies have shown that the HTKS is reliable and valid and significantly predicts academic achievement in diverse groups of children in the US, Asian, and European countries (e.g., Da Silva et al., 2025; Gonzales et al., 2021; McClelland et al., 2007, 2014; Spiegel and Lonigan, 2018; Von Suchodoletz et al., 2013) and recently in adolescents (Fernandes et al., 2023).

Nonetheless, studies reporting exploratory and/or confirmatory factor analyses of the original Head-Toes-Knees-Shoulders (HTKS) task remain limited. In the original validation study by Ponitz et al. (2008), the HTKS task was evaluated primarily through reliability and validity analyses. The authors examined internal consistency, inter-rater reliability, construct validity (via correlations with executive function and academic measures), and predictive validity. The HTKS was treated as a single composite measure of behavioral self-regulation. However, the study did not include exploratory or confirmatory factor analyses, nor did it report factor loadings or structural model fit indices. Similarly, McClelland et al. (2014) examined the psychometric properties and predictive validity of the HTKS but did not conduct exploratory factor analysis or report on its latent structure. Instead, the focus was on construct validity and predictive associations with academic outcomes. Indeed, a meta-analysis of the HTKS emphasized its strong predictive validity and reliability across studies, again focusing on correlational evidence and overall task performance rather than on exploratory or confirmatory factor structure (Kenny et al., 2023). Only recently, Ahmadi et al. (2025) examined the psychometric properties of the HTKS task in young Iranian children. Confirmatory factor analyses showed that both one-factor and two-factor structures were acceptable and that a one-factor solution was optimal.

To our knowledge, only one study—conducted by Cerino et al. (2018)—examined the psychometric properties of the HTKS in older adults, but it did not investigate its factorial structure. In this study, the HTKS was administered to a community-based sample of 150 older adults (aged 60 and above) living in Oregon, United States. Participants completed a battery of demographic and health-related self-report measures, including a screening item targeting memory-related concerns. Notably, individuals with chronic health conditions such as arthritis, cardiovascular disease, or diabetes were not excluded from participation, thereby enhancing the ecological validity of the sample by reflecting the typical health diversity of community-dwelling older adults. In addition to the HTKS, participants completed several standardized cognitive assessments from the NIH Toolbox Cognition Battery (NIHTB-CB), which included tasks measuring attention and cognitive flexibility (Dimensional Change Card Sort), inhibitory control (Flanker Inhibitory Control and Attention Test), working memory (List Sort Working Memory), and processing speed (Pattern Comparison Processing Speed Test). Emotional functioning was also assessed using the Positive and Negative Affect Schedule (PANAS), offering a multidimensional view of participants' affective states. Together, these measures allowed for a comprehensive evaluation of the HTKS's validity in capturing executive function capacities in older adults. The findings from Cerino et al. (2018) revealed that the HTKS task exhibited good internal consistency (Cronbach's α = 0.84). Correlational analyses revealed modest but statistically significant associations between HTKS performance and measures of processing speed (*r* = 0.24, *p* < 0.01) as well as attentional control assessed by the Dimensional Change Card Sort (DCCS; *r* = 0.17, *p* < 0.05). In contrast, no significant correlations were observed between HTKS scores and measures of working memory or affective functioning, lending support to the task's discriminant validity. Regression analyses further indicated that both attentional and inhibitory control significantly predicted HTKS completion time, even after controlling for potential confounding variables such as age, health status, and processing speed. Importantly, neither positive nor negative affect demonstrated significant relationships with HTKS performance or task completion time, suggesting that the task predominantly captures core executive functions rather than affective processes.

Despite promising preliminary findings, research on the application of the Head-Toes-Knees-Shoulders (HTKS) task in adult and geriatric populations remains very limited. Initial evidence indicates that older adults can successfully engage with the task and that HTKS performance is modestly associated with other indicators of cognitive functioning, including non-verbal reasoning and global cognitive performance (Cerino et al., 2018). However, comprehensive psychometric evaluations of the HTKS in non-child populations are notably scarce. Therefore, the current study aims to address this gap by evaluating the psychometric properties of the HTKS task in a sample of cognitively healthy Greek older adults. More specifically, based on the study of Cerino et al. (2018), the aims of the study were: (a) the preliminary test of the uni-factorial structure of the HTKS task, (b) the evaluation of its internal consistency reliability, (c) the test of the convergent/discriminant validity of the Greek version of the HTKS task with the Greek versions of the Mini-Mental State Examination (MMSE; Folstein et al., 1975), the Raven's CPM (CPM; Raven et al., 2004), the Gustafsson's Number Test (Gustafsson, 1984), the Positive and Negative Affect Scale (PANAS; Watson et al., 1988), the Barratt Impulsiveness Scale (BIS-11;Patton et al., 1995) and the Impulsive Behavior Scale (UPPS-P) and (d) the examination of the pattern of relationships among HTKS, CPM, Gustafsson's Number Series Test and Panas Positive and Negative Affect Schedule Scales.

## 3 Method

### 3.1 Participants

Although the number of the possible participants, that were initially examined via the Mini-Mental State Examination (MMSE; Folstein et al., 1975), comprised 156 older adults aged 65–90 years, the final sample of the present study consisted of 88 cognitively healthy older and overaged adults with adequate global cognitive functioning as indicated by the Mini-Mental State Examination scores. According to their performance on the MMSE, none of the 88 participants met diagnostic criteria for possible dementia, neither for mild cognitive impairment nor subjective cognitive impairment. Additional exclusion criteria were history of neurological conditions or psychiatric diseases, alcohol or drug abuse, severe head trauma, profound visual impairments, and verbal incomprehension.

Of the 88 participants, 76 (86.4%) were aged between 65 and 80 years (new-old adults), while the remaining 12 (13.6%) were between 81 and 88 years old (old-old adults). The mean age of the sample was 73.39 years. With regard to gender distribution, 48 participants (54.5%) were women and 40 (45.5%) were men. Educational level was categorized into three groups based on years of formal education: (a) 0–9 years, (b) 10–12 years, and (c) 13 or more years. Specifically, 29 participants (33.0%) had 0–9 years of education, 28 (31.8%) had 10–12 years, and 31 (35.2%) had 13 or more years of education.

Participants' professional background was classified into six categories:

- Scientific professionals (e.g., lawyers, physicians, engineers; 8.0%, *N* = 7)
- Scientific employees in the public or private sector (6.8%, *N* = 6)
- Employees (e.g., salespersons, secretaries, technicians; 40.9%, *N* = 36)
- Entrepreneurs, professionals, and craftsmen (10.2%, *N* = 9)
- Farmers and stockbreeders (10.2%, *N* = 9)
- Homemakers (23.9%, *N* = 21)

The participants were recruited using a convenience sampling method from various regions of Greece and their place of residence was classified into three categories: village (24.0%, *N* = 21), town (4.5%, *N* = 4), and city (71.6%, *N* = 63).

### 3.2 Measurements

#### 3.2.1 Head-Toes-Knees-Shoulders

The Head–Toes–Knees–Shoulders task (HTKS; McClelland et al., 2007; Ponitz et al., 2008, 2009) is a brief, game-like measure of behavioral self-regulation that integrates executive functions, including working memory, inhibitory control, attentional focus and cognitive flexibility. The assessment consists of 30 items across three sections with increasing rule complexity. Participants are instructed to perform the opposite of a spoken command (e.g., touch head when instructed to touch toes), with later sections requiring the use and switching of four rule pairs (head–toes; shoulders–knees). Administration takes approximately 5–7 min and requires no materials. Responses are scored on a 3-point scale (0 = incorrect, 1 = self-corrected, 2 = correct), yielding total scores from 0 to 60, with higher scores indicating better self-regulation. The HTKS is available at no cost for research purposes through an online request at http://health.oregonstate.edu/labs/kreadiness/resources, and completion of required online training is necessary before use.

#### 3.2.2 Mini-Mental State Examination

The Mini-Mental State Examination (MMSE; Folstein et al., 1975) was used to assess global cognitive functioning and to determine eligibility for study participation. The MMSE is a brief screening instrument consisting of six subtests with 19 items that evaluate orientation to time and place, naming, memory, attention, comprehension, praxis, and the ability to follow verbal and written commands. Administration requires approximately 5–10 min, with a maximum possible score of 30 and no time limit. The Greek version of the MMSE, translated and standardized by Fountoulakis et al. (2000), was administered. According to the Greek version of the MMSE, total scores of 23 or lower indicate cognitive impairment into stages of dementia, a cut-off that is widely accepted and applied in clinical and research settings (Creavin et al., 2016). Scores of 24–25 suggest probable mild cognitive impairment (Bartos and Raisova, 2016; O'Bryant et al., 2008), and scores of 26–27 are indicative of subjective cognitive impairment. In the present study, older adults scoring below 28 were excluded, and only participants with scores ranging from 28 to 30 were included, indicating intact cognitive functioning.

#### 3.2.3 Raven's educational—Colored Progressive Matrices

The Colored Progressive Matrices (CPM: Raven et al., 2004) is a nonverbal measure of general cognitive ability based on Spearman's (1904) *g* factor. The CPM consists of 36 items divided into three subscales (A, AB, and B) of increasing difficulty. Each item presents an incomplete pattern with six response options, one of which correctly completes the pattern. Scores range from 0 to 36, and administration is untimed. Although primarily developed for children aged 4–11 years, the CPM has been used with older adults, special populations, and linguistically diverse samples. The Greek standardized version of the CPM was administered (Sideridis et al., 2015). The Greek version of CPM has also been tested in young children and older adults (Papantoniou et al., 2015). The internal consistency reliability in the current sample was Cronbach's *a* = 0.88.

#### 3.2.4 Number Series test

Number Series (NS: Gustafsson et al., 1981) test is a general cognitive ability (intelligence) test and it measures inductive reasoning, which is a key component of fluid intelligence. Gustafsson et al.'s (1981) and subsequent work (Gustafsson, 1984) used this test to analyze the hierarchical structure of intellectual abilities, particularly focusing on the relationship between general intelligence (*g*) and fluid intelligence and found —applying structural equation modeling (SEM)— fluid intelligence to be identical with general cognitive ability (*g*). The test consists of 20 numerical problems (items), each presenting a series of 5 or 6 numbers and the task is to add two more numbers to the series. The test score is the sum of correct answers, ranging from 0 to 20 points. The test has a time limit of four (4) minutes. The psychometric properties of the Greek version of NS have been tested by Efklides et al. (1999). In the present study, Cronbach's alpha reliability coefficient was 0.82.

#### 3.2.5 Positive and Negative Affect Schedule

The Positive and Negative Affect Schedule (PANAS; Watson et al., 1988) is a 20-item self-report measure assessing positive and negative affect. The scale comprises two subscales—Positive Affect and Negative Affect—each consisting of 10 emotion descriptors (e.g., proud, active, hostile, irritable). Items are rated on a 5-point Likert scale ranging from 1 (very few times or not at all) to 5 (too many times). In the present study, the Greek version of the scale assessing affect over the previous 2 weeks, translated and adapted by Moraitou and Efklides (2009), was administered. Previous research has demonstrated that the Greek PANAS replicates the original two-factor structure (Papantoniou et al., 2012). Internal consistency reliability in the current sample was acceptable (Cronbach's α = 0.79 for Positive Affect and α = 0.77 for Negative Affect).

#### 3.2.6 The Barratt Impulsiveness Scale (BIS-11)

The BIS-11 (11th version) is the latest version of the BIS (Patton et al., 1995) and is designed to measure the behavioral or personality trait of impulsivity. The instrument consists of 30 items describing common impulsive and nonimpulsive behaviors. The nonimpulsive items are reverse scored so that higher scores indicate greater impulsive behavior (Vasconcelos et al., 2012). Items are rated on a 4-point Likert scale ranging from rarely/never to almost always/always. Total scores range from 30 to 120, with higher scores indicating greater levels of impulsivity. The BIS-11 yields three subscale scores–attentional impulsiveness, motor impulsiveness, and nonplanning impulsiveness–and demonstrates good overall internal consistency (Cronbach's α = 0.83). In the present study, the Greek version of BIS-11, translated by Giotakos et al. (2003) and adapted by Tsatali et al. (2021), was administered. The overall internal consistency reliability of the BIS-11 in the present study was good (α = 0.81). Internal consistency estimates for the subscales were α = 0.61 for attentional impulsiveness, α = 0.76 for motor impulsiveness, and α = 0.73 for nonplanning impulsiveness, consistent with findings reported by Tsatali et al. (2021).

#### 3.2.7 The Impulsive Behavior Scale (UPPS-P)

The UPPS-P Impulsive Behavior Scale was originally developed by Whiteside and Lynam (2001) and consists of 59 items. In the present study, however, the short form of the UPPS-P was administered because it requires less time to complete while retaining satisfactory psychometric properties (Cyders et al., 2014) compared to the original version. The short UPPS-P is a 20-item measure derived from the Five-Factor Model of personality (Lynam, 2013) and assesses impulsive dispositions across five lower-order traits identified in the structural model proposed by Whiteside and Lynam (2001): (a) emotion-based rash action (including negative and positive urgency), (b) sensation seeking, and (c) deficits in conscientiousness, reflected in lack of premeditation and lack of perseverance.

The Greek version of the UPPS-P used in the present study was translated and adapted by Tsatali et al. (2021). This version comprises three factors: (a) deficits in conscientiousness, (b) emotion-based rash action, and (c) sensation seeking. Items are rated on a four-point Likert-type scale ranging from 1 (strongly agree) to 4 (strongly disagree). Items corresponding to the second and third factors are reverse scored. Higher total scores reflect greater impulsive behavior (Cyders et al., 2014). In the present study, the overall internal consistency reliability was good (Cronbach's α = 0.77). Cronbach's alpha was 0.72 for the first factor (deficits in conscientiousness), 0.75 for the second factor (emotion-based rash action), and 0.69 for the third factor (sensation seeking). The findings of the present study are consistent with those reported by Tsatali et al. (2021).

### 3.3 Procedure

For the purposes of the present study, permission to use the Head-Toes-Knees-Shoulders (HTKS) measure was obtained from the developers and Oregon State University after they were informed about the aims of the research. Following this communication and approval, the administration guidelines were provided. The data collection team, consisting of two of the authors (post-graduate students), completed formal training and successfully passed an online certification assessment for the administration of the HTKS. Upon successful completion, both researchers received official certification confirming their qualifications to administer the measure. In addition, they received training in the administration of all other instruments included in the study.

Participants were recruited from all over Greece (via an invitation to participate in research about a new measure that estimates the behavioral self-regulation of older adults). The circulation and the information about the aforementioned invitation—with the consequence of the random data collection from all over Greece—was conducted by students from the University of Ioannina in Greece during their attendance and research exercises of two introductory courses of Psychology.

Participants were provided with a detailed information sheet and a written informed consent form prior to participation. The consent form included demographic items (e.g., place of residence, gender, age, educational level, previous occupation, and general health information). The information sheet also contained the research team's contact information (telephone number and email address), and participants were encouraged to seek clarification regarding any aspect of the study if needed.

Data were collected individually either at the Psychology Laboratory of the Department of Early Childhood Education in the University of Ioannina or, when participants were unable to be assessed at the laboratory, in their own settings. In all cases, the psychometric measures were administered in a quiet environment with minimal external distractions. Given the number of instruments included in the study protocol, administration was completed across two individual sessions scheduled on separate days to reduce potential fatigue effects. Each session had a duration of approximately 35–40 min. To control for potential order effects, instruments were administered in a randomized sequence in each of the two sessions.

The study protocol adhered to the ethical principles outlined in the Helsinki Declaration and received approval from the Scientific and Ethics Committee of the University of Ioannina (37008/2023).

### 3.4 Data analysis

Exploratory factor analysis (EFA) was conducted in the current study —with the use of IBM SPSS Statistics, version 25—to examine the factorial structure of the Greek version of the HTKS in a sample of cognitively healthy Greek adults aged 65 years and older. The rest of the statistical analyses—which are a calculation of Cronbach's alpha internal consistency reliability coefficients and Pearson's *r* correlation coefficients examining the associations between the variables under analysis and the convergence of HTKS with respect to other instruments—were also performed, with the use of IBM SPSS Statistics, version 25 and the statistical significance was set at 0.05. Similarly, since path analysis evaluates more precisely the causal relationships among the examined variables (Bentler, 2005; Brown, 2006), in the present study path analysis was preferred over multiple regression analysis and was conducted in the statistical program EQS 6.1. (Bentler, 2005). All variables were treated as observed composite scores, and parameters were estimated using maximum likelihood based on the sample covariance matrix. Model fit was evaluated using χ^2^, CFI, RMSEA, and SRMR.

## 4 Results

### 4.1 Factor structure of the Head-Toes-Knees-Shoulders

Because of the small size of the sample, we proceeded by conducting an exploratory factor analysis (EFA), without rotation, on the three (3) total scores (measured variables) reflecting the measurements of participants across the three (3) sets of the HTKS. The Kaiser-Mayer-Olkin measure was applied to evaluate the total sample suitability, the value of which was K.M.O = 0.69. Barlett's sphericity control was statistically significant χ^2^ = 89.742, df = 3, *p* < 0.001. Kaiser's criterion (eigenvalue > 1.0) was applied to determine the number of factors to be retained. The analysis yielded a single factor with an eigenvalue greater than 1.0. This factor had an eigenvalue of 2.185 and accounted for 72.83% of the total variance. The total scores of the measurements appeared to load on a single factor (Table 1).

**Table 1:** Exploratory factor analysis of the Head-Toes-Knees-Shoulders task based on the 3 total scores reflecting the measurements of participants across the 3 sets of the HTKS.

Variable	Factor 1
HTKS total set 1	0.813
HTKS total set 2	0.890
HTKS total set 3	0.856

### 4.2 Internal consistency reliability of the Head-Toes-Knees-Shoulders

Cronbach's alpha coefficient was calculated to assess the internal consistency reliability of the HTKS. In the present sample of older adults, internal consistency reliability was acceptable to good (α = 0.79).

### 4.3 Convergent validity of the Head-Toes-Knees-Shoulders HTKS

The convergent validity of the Greek version of the HTKS was examined using Pearson correlation coefficients (*r*) between HTKS and the other instruments administered in the study. A positive and statistically significant moderate correlation was observed between HTKS and CPM (*r* = 0.438, *p* < 0.01), as well as between HTKS and the Gustafsson Number Series Test (*r* = 0.433, *p* < 0.01). In addition, a positive and statistically significant but small correlation was found between HTKS and MMSE (*r* = 0.263, *p* < 0.01). In contrast, HTKS was not significantly correlated with Positive Affect (PA) or Negative Affect (NA) as measured by the PANAS. Furthermore, no significant correlations were found between HTKS and any of the three factors of the BIS-11 or the UPPS (Table 2).

**Table 2:** Pearson's correlations between the Head-Toes-Knees-Shoulders task and the instruments used in the study.

Variable	HTKS
HTKS	1.000
CPM total	0.438^**^
MMSE total	0.263^*^
Number series test	0.433^**^
PANAS Positive	0.180
PANAS negative	0.095
BIS nonplanning impulsiveness	−0.006
BIS motor impulsiveness	−0.026
BIS attention impulsiveness	−0.090
UPPS deficits in conscientiousness	−0.064
UPPS emotion-based rash action	−0.079
UPPS sensation seeking	−0.067

^**^The symbol indicates statistical significance at the 0.01 level (two-tailed).

^*^The symbol indicates statistical significance at the 0.05 level (two-tailed).

### 4.4 Relationships among behavioral self-regulation/executive functioning (HTKS), nonverbal cognitive ability (CPM), general intelligence (NF) and affect (PANAS)

To further evaluate the relationships between behavioral self-regulation / executive functioning (HTKS) and nonverbal cognitive ability (CPM) and general intelligence (NF), on the one hand, as well as between behavioral self-regulation / executive functioning (HTKS) and positive and negative affect (PANAS), on the other, a path analysis was conducted in EQS Version 6.1. (Bentler, 2005) using the maximum likelihood estimation procedure and performed on the covariance matrix, derived from the full sample (*N* = 88). Because of the relatively small sample size of the group, the covariance matrix was based on total scores (measured variables) of the measures. In the equations of the path analysis, the performances of the participants in CPM and NS test were defined as independent variables (exogenous) and the performances of the participants in HTKS, PANAS Positive and PANAS Negative affect as dependent variables (endogenous variable). Model fit was assessed using the chi-square (χ^2^) test, where a non-significant result indicates that the hypothesized model adequately reproduces the observed variance–covariance structure (Brown, 2006; Kline, 2003). The χ^2^/df ratio –which was introduced by Wheaton et al. (1977) in attempt to use χ^2^ in a manner that would foster more realistic model evaluation– was also calculated. Given the test's sensitivity to sample size, additional fit indices were employed. The Root Mean Square Error of Approximation (RMSEA) –which, according to Fan and Sivo (2007), stood out as having ideal behavior patterns expected of good model fit index– was used to evaluate how well the model would fit the population covariance matrix. According to established guidelines, RMSEA values ≤ 0.05 indicate a close fit, values between 0.05 and 0.08 suggest a reasonable fit, and values ≥ 0.10 indicate poor fit (Brown and Cudeck, 1993; Kline, 2003). Furthermore, CFI values between 0.95 and 1.00 reflect a good fit, while values between 0.90 and 0.95 represent an acceptable fit (Bentler, 1990; Brown, 2006; Schweizer, 2010). Lastly, the Standardized Root Mean Square Residual (SRMR), which reflects the average discrepancy between observed and predicted correlations, was also examined. An SRMR value below 0.08 is considered indicative of a good model fit (Bentler, 2005; Brown, 2006; Hu and Bentler, 1999).

The fit indices which were provided from the application of the path analysis for the proposed model met all the above-mentioned corresponding criteria [χ^2^ (4) = 0.494, *p* = 0.974, χ^2^/df = 0.123 CFI = 1.000, SRMR = 0.020, and RMSEA = 0.000 (CI90%: cannot compute boundary of Confidence Interval)]. Furthermore, all parameters were statistically significant (*p* < 0.05) except for behavioral self-regulation/executive function's loading (0.180) on positive affect (*p* = 0.088 and *z* value = 1.708). This parameter, marginally dropped (χ^2^ = 2.917) in the process of the Wald test since χ^2^ ≥ 3.000, suggests a reasonable significance of the parameter that is included in the model (Brown, 2006).

**Figure 1:**
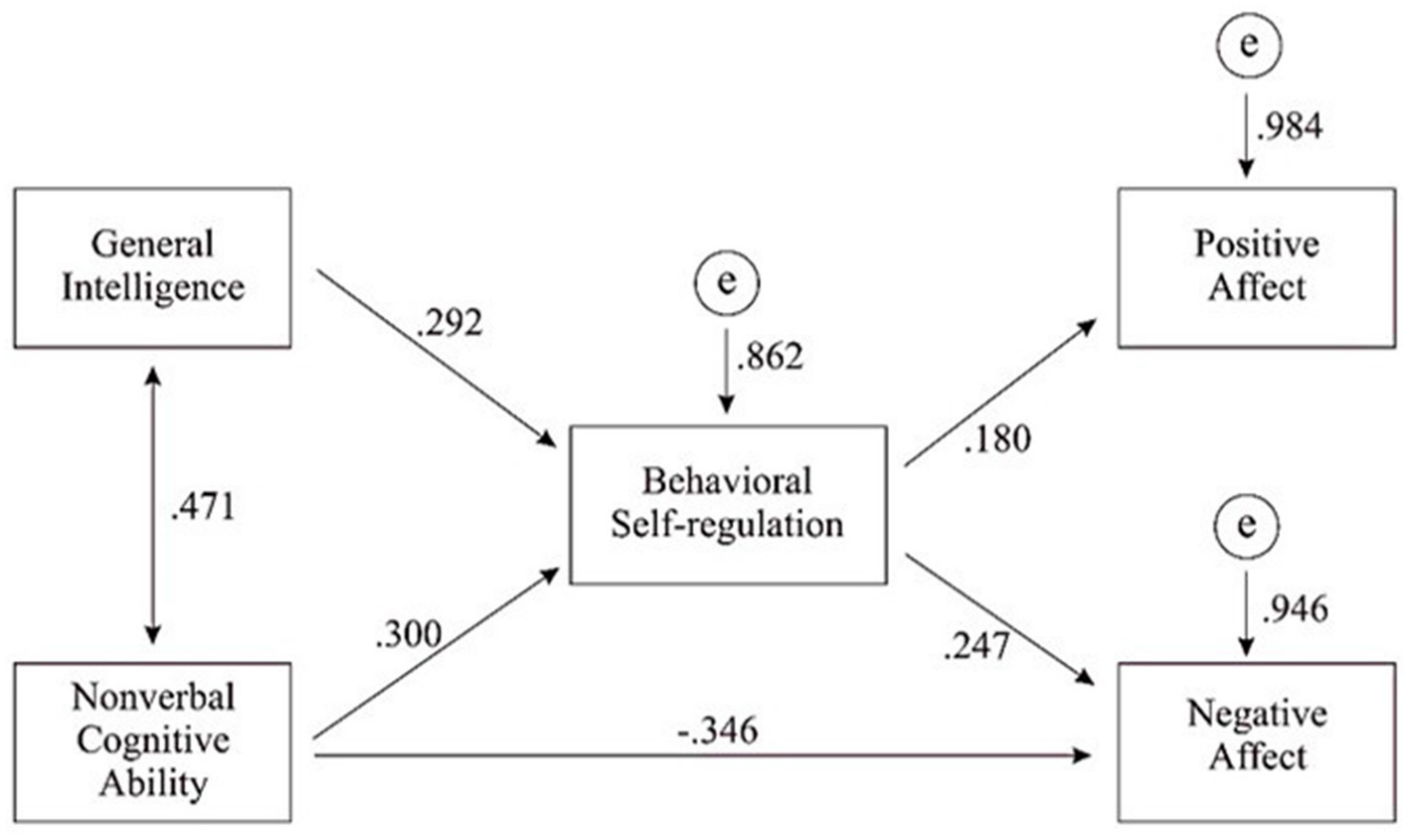
Path diagram illustrating the relationships among behavioral self-regulation, nonverbal cognitive ability, general intelligence, and affect (standardized solution). e, Error variance of variable. All loadings and parameters presented are statistically significant (*p* < 0.05) except for behavioral self-regulation's loading on positive affect (*p* = 0.088).

The proposed path model demonstrated good fit to the data, indicating that the model adequately reproduced the observed covariance matrix. Standardized estimates showed that both nonverbal cognitive ability and general intelligence were positively associated with behavioral self-regulation (performance on HTKS), jointly explaining 25.8% of its variance (*R*^2^ = 0.258). Behavioral self-regulation (performance on HTKS) was found to have a non-significant low positive effect on positive affect, explaining 3.24% of its variance (*R*^2^ = 0.032). Negative affect was positively associated with the performance on HTKS and negatively affected by nonverbal ability, with the two cognitive variables accounting for 10.6% of its variance (*R*^2^ = 0.106). Performance on CPM and NS were moderately and positively correlated (*r* = 0.471), reflecting shared variance between the two cognitive ability measures (Figure 1).

## 5 Discussion

The Head-Toes-Knees-Shoulders (HTKS) is a children's behavioral self-regulation/executive function assessment task, which integrates the measurement of executive functions (working memory, inhibitory control, cognitive flexibility) into a brief task resembling a game. The aim of the present study was to examine some psychometric properties (factorial structure, internal consistency reliability and convergent validity) of the Greek version of the HTKS in a sample of Greek older adults' population. Additionally, a path analysis was conducted to further examine, in older adults, the pattern of relationships among behavioral self-regulation/executive functioning, nonverbal cognitive ability, general intelligence, positive and negative affect.

Initially, the aforementioned EFA results revealed a unifactorial structure for the Greek version of the HTKS behavioral self-regulation measure in a sample of Greek older adults aged 65 and over. Although the factor structure has not been yet fully examined by the original developers of the instrument, these findings support their proposal to calculate a single composite score of behavioral self-regulation. Moreover, the results are consistent with those of Ahmadi et al. (2025), who also identified a one-factor solution as optimal, albeit in a sample of young children.

With respect to the internal consistency reliability of the Greek version of the HTKS, acceptable reliability was demonstrated, as the 30 items of the task showed adequate internal consistency in the present sample (α = 0.79), a finding that is consistent with the findings of Cerino et al. (2018) in a sample of older adults too.

Concerning Panas Positive and Negative Affect Schedule Scales, the Pearson correlation analysis indicated negligible to weak, not statistically significant, correlations with the HTKS scores. This result is consistent with the findings of Cerino et al. (2018), who found no correlation between affective state, as measured by PANAS and HTKS, suggesting that the HTKS primarily assesses executive functions such as attention and inhibitory control rather than affect.

Similarly, no statistically significant correlation was found between HTKS performance and impulsivity, which was assessed using both the BIS-11 and the UPPS-P scales. Admittedly, impulsivity is often regarded as a consequence of impaired executive functioning, particularly when dysfunctional inhibitory processes and strong impulses co-occur (Portugal et al., 2018). Despite some overlap, executive functions and impulsivity are phenotypically and genetically distinct constructs, with only weak correlations observed between self-report measures of impulsivity—as the BIS-11 and the UPPS-P scales—and laboratory-based executive function tasks—as the HTKS task—(Stautz et al., 2016), a pattern consistent with our findings. Furthermore, given that impulsivity comprises multiple facets, its conceptual homogeneity has been increasingly questioned (Stahl et al., 2014).

Additionally, Pearson correlation analysis revealed moderate, positive and statistically significant correlations between HTKS and global cognitive functioning (MMSE), nonverbal cognitive ability (CPM), and fluid intelligence/general intelligence (NS test) indicating a good convergent validity of HTKS as a behavioral self-regulation/executive function task discrete from cognitive ability tasks. Regarding MMSE, the finding may be explained by the fact that both measures overlap in cognitive domains, such as attention, memory and motor planning (Ghawami et al., 2019; Gómez-Soria et al., 2021). With respect to nonverbal cognitive ability (CPM) and fluid intelligence/general intelligence (NS test), moderate, positive, and statistically significant correlations were found with HTKS performance. The findings are consistent with neuropsychological evidence in older adults showing a moderate association between executive functions and Raven's Progressive Matrices performance (Kessels et al., 2009). Similarly, in the study of Friedman et al. (2006) working memory—conceptualized as a core component of executive function—demonstrated high and statistically significant correlations with general intelligence. This relationship reflects the ability to manage and update information stored in working memory, a skill that is essential for successful performance on general intelligence tests. Additionally, inhibitory control and cognitive flexibility showed statistically significant but smaller correlations with general intelligence. Besides, Barbey et al. (2012) provided empirical support for a shared neuroanatomical substrate underlying general intelligence and executive functions, thereby reinforcing the view that these constructs rely on an integrated and distributed network of brain regions rather than isolated neural loci. Furthermore, the moderate, positive, and statistically significant correlation of fluid intelligence with executive functions has been confirmed in several studies (e.g., Ackerman et al., 2005; Kane et al., 2004; Unsworth et al., 2014). For example, Van Aken et al. (2016) investigated the relationship between these two constructs. Their results showed a statistically significant positive correlation between fluid intelligence and executive functions, especially working memory. Executive functions represent a broad set of higher-order cognitive processes, including working memory, task switching, attentional control, and response inhibition (Diamond, 2013; Lezak et al., 2012; Stuss and Knight, 2013). Because fluid intelligence encompasses abilities such as short-term memory, processing speed, and various forms of reasoning (e.g., quantitative, verbal, inductive, and deductive), it closely aligns with neurocognitive conceptualizations of executive functioning (Fisher et al., 2019). Moreover, some researchers propose that declines in information processing speed may drive age-related changes in a range of neurocognitive capacities, including executive functions (Diamond, 2013; Rozas et al., 2008).

The aforementioned moderate and positive relationships between behavioral self-regulation/executive functions, and nonverbal cognitive ability, and fluid intelligence/general intelligence were also found in path analysis which revealed that both nonverbal cognitive ability and fluid intelligence/general intelligence positively affect behavioral self-regulation/executive functions jointly explaining 25.8% of its variance, underscoring the link between higher-order cognitive ability and behavioral self-regulation in later life. This pattern aligns with prior evidence indicating that fluid intelligence—operationalized here through CPM and NS test—and executive functioning are interrelated constructs in older adulthood (Barbey et al., 2012; Fisher et al., 2019; Friedman et al., 2006; Kessels et al., 2009). Nevertheless, a meaningful proportion of variance in the performance on HTKS remained unexplained by measures of cognitive ability. This finding may reflect the distinction between the cognitive demands assessed by fluid reasoning (inductive) tasks and those required for behavioral regulation. Fluid reasoning tasks primarily engage core executive processes, such as working memory and inhibition. In contrast, behavioral regulation tasks also involve real-time response suppression, motor coordination, selective and divided attention, and the continuous implementation of rules under performance demands (Verhaeghen and Cerella, 2002).

Additionally, the path analysis indicated the statistically non-significant correlation between behavioral self-regulation/executive functioning and positive affect, consistent with the findings of Cerino et al. (2018) and Berk et al. (2017). The non-significant low positive effect of behavioral self-regulation/executive functions on positive affect suggests that behavioral self-regulation may possibly contribute to emotional well-being in later life, beyond cognitive ability (Idowu et al., 2024). This finding is consistent with the view that emotional experience and emotion regulation follow a more complex developmental trajectory and cannot be explained solely by cognitive performance (Isaacowitz, 2022). Research emphasizes that executive functions (including inhibitory and attentional control) decline with age, but emotion regulation and affective processes can follow distinct life-span trajectories (Löckenhoff and Carstensen, 2004). The differentiation between general cognitive ability and executive/self-regulatory processes is consistent with evidence that executive functions in older adulthood are multidimensional and show partially distinct relationships with emotional functioning (Idowu et al., 2024).

In our study, although the Pearson's bivariate correlation between performance on HTKS and negative affect was minimal, non-significant and consistent with the finding of Cerino et al. (2018), the path model—in which performance on cognitive ability measures and HTKS were included as predictors—revealed a statistically significant moderate negative direct effect of nonverbal cognitive ability on negative affect and a statistically significant low positive direct effect of behavioral self-regulation on negative affect, despite of the positive association between performance on CPM and HTKS.

The discrepancy between the non-significant bivariate correlation involving negative affect and the subsequent path analysis can be explained by the fact that path analysis decomposes zero-order correlation into direct and indirect effects. As a result, an association that appears non-significant at the bivariate level may emerge as a direct effect once other pathways are modeled (Wooldredge, 2021). This is often interpreted as adjustment effect rather than mediation (McCrea, 2014).

Therefore, the unique relationship (direct low positive effect) between performance on HTKS and negative affect was positive and became statistically more pronounced. This latter finding may be explained by evidence that inhibitory self-control efforts temporarily raise negative affect (Wenzel et al., 2024). Conceptually, this may reflect that in older adults, behavioral self-regulation, measured by tasks like the HTKS, may incorporate aspects of effortful engagement and inhibitory monitoring that are statistically independent from general cognitive ability, which may in turn relate to negative emotional experience. One of the most consistent findings in the study of emotion and aging is that older adults experience positive affect and at least an equal, if not a lesser, amount of negative affect than do their younger counterparts (Doerwald et al., 2016; Tsai et al., 2018).

Prior research adapting the HTKS for older adults demonstrates that the measure captures executive components that are empirically separable from affective constructs (Cerino et al., 2018). The present findings extend this work by showing that when examined in a multivariate framework, behavioral self-regulation exhibits distinct associations with both positive and negative affect beyond shared variance with cognitive ability.

Some limitations of the present study should be acknowledged. First, the sample of participating older adults was relatively small and insufficient to support conducting both confirmatory factor analysis of the HTKS and latent variable path analysis. Further research with a larger sample is warranted to validate the factor structure of the HTKS in the Greek older adult's population and its associations with cognitive and affective variables as well. Second, the MMSE has limitations as a standalone cognitive screening instrument. Its performance is substantially influenced by demographic and educational factors (Santana et al., 2016), it is susceptible to ceiling effects (Friedman et al., 2012), and it provides limited assessment of executive functioning (Kessels et al., 2009). Consequently, its sensitivity to subtle cognitive impairments is reduced (D'Ignazio et al., 2025), as individuals scoring within the normal range (28–30) may nevertheless exhibit measurable cognitive deficits (Friedman et al., 2012). Future research should incorporate more sensitive screening measures, such as the Montreal Cognitive Assessment (MoCA; Nasreddine et al., 2005), which has consistently demonstrated enhanced sensitivity and diagnostic accuracy for detecting early and preclinical cognitive decline, thus ensuring a more precise characterization of participants' cognitive status (Ciesielska et al., 2016; Jia et al., 2021). Third, the study did not include additional measures of executive functioning, such as working memory, inhibitory control, attentional control, and cognitive flexibility. Examining the associations between these measures and HTKS performance would provide a more comprehensive understanding of behavioral self-regulation in older adults and strengthen evidence for the instrument's discriminant validity. Furthermore, executive functioning also encompasses more emotionally charged processes (e.g., decision-making, motivational processing, and emotion regulation). The inclusion of measures assessing these “hot” executive functions would offer a clearer and more comprehensive picture of how cognitive processes interact with and influence emotional functioning in older adults. Future research should therefore incorporate validated assessments of “hot” executive functions in order to examine how these processes relate to HTKS. Fourth, although the association between neuropsychological outcomes and mood and affective symptoms in aging has been established (Ong et al., 2022; Toh et al., 2020; Wyman et al., 2022; Yagi et al., 2020), this pattern was not replicated in the present study, as the observed associations with affective outcomes were weak, and their interpretation remains tentative. Additionally, motivation influences performance (Vallet et al., 2020), and performance validity measures can yield false positives or be affected by demographic factors (Davis, 2018). As such, further research including a more comprehensive evaluation of emotional functioning is needed to better disentangle their association with cognitive performance in older adults. Furthermore, differences in educational background (e.g., greater or lesser familiarity with standardized testing) may introduce test bias, and lower levels of education may negatively affect cognitive test performance independently of underlying cognitive impairment (Reyes et al., 2024). Therefore, including participants' educational attainment as a control variable in future research would help account for its potential confounding effects on cognitive performance and improve the validity of study findings (Kessels and Hendriks, 2023). Finally, educational attainment has been proposed to enhance cognitive reserve—a person's ability to maintain cognitive functioning despite age-related or pathological changes in the brain—thereby serving as a protective factor against the effects of neuropathology on cognition, such as delaying the onset of cognitive impairment (Stern et al., 2020). Hence, cognitive reserve could also be included as a crucial factor in future studies.

Notwithstanding its limitations, the present study represents the first empirical effort to adapt the HTKS in the Greek older adult's population. The results provide preliminary evidence of the instrument's feasibility, initial construct-related validity and potential applicability for examining behavioral self-regulation in Greek older adults.

## Data Availability

The raw data supporting the conclusions of this article will be made available by the authors, without undue reservation.
